# Protocol for a systematic review and network meta-analysis of randomised controlled trials examining the effectiveness of early parenting interventions in preventing internalising problems in children and adolescents

**DOI:** 10.1186/s13643-020-01500-9

**Published:** 2020-10-19

**Authors:** Ilaria Costantini, Elise Paul, Deborah M. Caldwell, José A. López-López, Rebecca M. Pearson

**Affiliations:** 1 Population Health Sciences, Bristol Medical School, University of Bristol, Oakfield House, Oakfield Grove, Bristol, BS8 2BP UK; 2 Department of Basic Psychology & Methodology, University of Murcia, Murcia, Spain

**Keywords:** Systematic review, Internalising problems, Parenting interventions, Network meta-analysis, Prevention, Randomised controlled trials

## Abstract

**Background:**

Internalising problems, such as depression and anxiety, are common and represent an important economical and societal burden. The effectiveness of parenting interventions in reducing the risk of internalising problems in children and adolescents has not yet been summarised. The aims of this review are to assess the effectiveness of parenting interventions in the primary, secondary and tertiary prevention of internalising problems in children and adolescents and to determine which intervention components and which intervention aspects are most effective for reducing the risk of internalising problems in children and adolescents.

**Methods:**

Electronic searches in OVID SP versions of MEDLINE, EMBASE and PsycINFO; Cochrane Central Register of Controlled Trials; EBSCO version of ERIC and ClinicalTrials.gov have been performed to identify randomised controlled trials or quasi-randomised controlled trials of parenting interventions. At least two independent researchers will assess studies for inclusion and extract data from each paper. The risk of bias assessment will be conducted independently by two reviewers using the Cochrane Collaboration’s Risk of Bias Assessment Tool. Statistical heterogeneity is anticipated given potential variation in participant characteristics, intervention type and mode of delivery, and outcome measures. Random effects models, assuming a common between-study variability, will be used to account for statistical heterogeneity. Results will be analysed using a network meta-analysis (NMA). If appropriate, we will also conduct a component-level NMA, where the ‘active ingredients’ of interventions are modelled using a network meta-regression approach.

**Discussion:**

Preventing and reducing internalising problems could have major beneficial effects at the economic and societal level. Informing policy makers on the effectiveness of parenting interventions and on which intervention’s component is driving the effect is important for the development of treatment strategies.

**Systematic review registration:**

International Prospective Register for Systematic Reviews (PROSPERO) number CRD42020172251

## Background

### Description of condition

Internalising problems, defined as symptoms and disorders of mood (e.g., depression, anxiety, somatic problems, obsessive and stress-related problems) represent a broad range of emotional disturbances. The study of internalising problems in young children has represented a challenge. One reason is that symptom manifestation varies greatly according to developmental stage, making internalising problems in children difficult to classify [1–3]. Another reason is that the acknowledgement of child psychopathology, particularly at younger ages, has been attained slowly from parents, clinicians and researchers and has been met with multiple criticisms and controversies [2–5]. However, despite these difficulties, important progress has been made over the last few decades in the identification and classification of internalising problems in children.

Depression and anxiety in childhood and adolescents are a leading cause of DALY (disability-adjusted life-year) losses in the Americas and in Europe [6–8]. In addition, the burden of non-communicable diseases is expected to increase in low- and middle-income countries due to better treatment of communicable diseases [6]. Internalising problems have a broad range of additional negative consequences on the individual and on society [9–12].

They are among the most common mental health disorders, with a high lifetime prevalence of anxiety and depression (28.8% and 20.8%, respectively) [13], which represent the two most common internalising problems. Previously assumed to have their onset in adolescence, there is substantial evidence that internalising problems can occur in children younger than age 5 [14, 15]. The prevalence estimates of internalising disorders in children aged 0–3 years old have been reported to be around 3%, with higher rates in low-income countries due to a greater presence of post-traumatic stress disorder [2, 5, 14–17].

Whilst there is evidence [18–22] for genetic heritability of depression and anxiety disorders, there is also clear evidence for the importance of environmental factors [19, 23]. The familial environment and the parent-child relationship are hypothesised to have important influences on child emotional development [24–27]. Parent characteristics such as low self-esteem and depressive symptoms, dysfunctional family environments and socioeconomic disadvantage are important risk factors for the development of child internalising disorders [25, 28, 29]. Specific negative parenting practices have been found to be associated with mood and anxiety symptoms [26, 27, 30–38]. For example, negative parenting such as intrusiveness, inter-parental conflict, inconsistent discipline and over-involvement are associated with anxiety and depression problems in the child [26, 27]. Conversely, there is evidence that positive parenting practices are associated with higher child self-esteem, better social adjustment and academic competence and may confer protective effects against stressors in later life and risk factors for mental health issues (e.g., substance misuse) [27, 39–41].

Growing evidence from different research fields has identified the period between birth (possibly even in utero [42, 43]) to 3 years old as a sensitive developmental period because of brain cell and synaptical growth, and motor, behavioural, linguistic and affective development [44–48]. Disruptions in this early critical phase of life may therefore have particularly detrimental consequences on later mental health. Investing in young children (particularly in pre-schoolers) could therefore provide the greatest rates of return to human investment [49]. We therefore hypothesise that early interventions that focus on changing parenting may be most effective in preventing later child internalising problems [49].

Given the evidence for a direct link between parental mental health and parenting behaviour [25], it is important to consider the role of parental mental health in the success of parenting interventions. In fact, parental mental health may influence child and adolescent mental health regardless of its impact on parenting practices. According to Bandura’s social learning theory [50], humans learn from one another via modelling, observation and imitation. Thus, a child may, for example, learn from a socially anxious parent to fear social situations and interactions. Similarly, a child may also appraise different cognitive biases that may sustain or provoke negative mood from a depressed or anxious parent [51]. Such behaviours may not be linked to parenting directly but rather observing the depressed and anxious behaviours of their parent in other situations (e.g., interaction with the partner or other family components, or outside the familial environment). It is therefore plausible that intervening on parenting only, without addressing parental manifestations of anxiety or depression, may be inadequate. We consequently explore the role of parental mental health either as a moderator or mediator of parenting interventions.

### Description of intervention

Given the important role of parenting for the risk of child internalising problems, our review will focus on parenting interventions.

In this review, parenting interventions refer to programmes that aim to prevent or treat children’s behavioural and emotional problems by improving family interactions, parenting behaviours and parents’ knowledge, attitudes and practices [52]. The transition to parenthood starts during pregnancy, and therefore, parenting interventions can occur both during the antenatal period and after delivery. Such interventions are typically short-term [53], and the timing of interventions varies considerably.

**Fig. 1:**
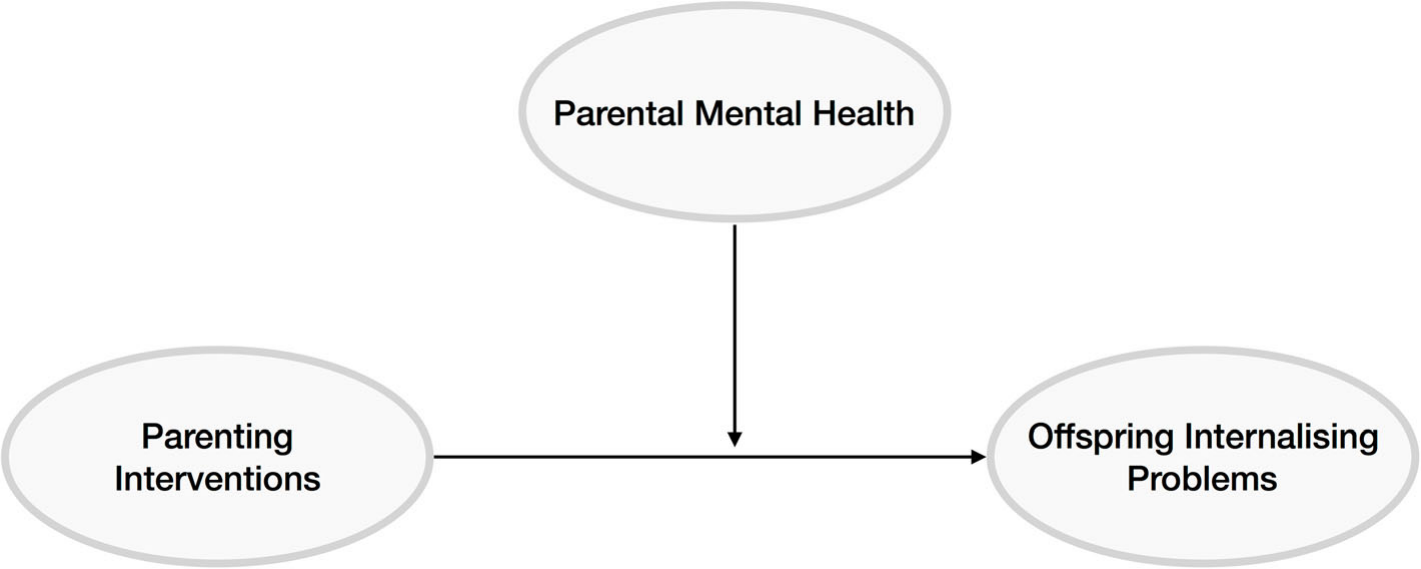
Parental mental health moderates the effectiveness of the intervention

**Fig. 2:**
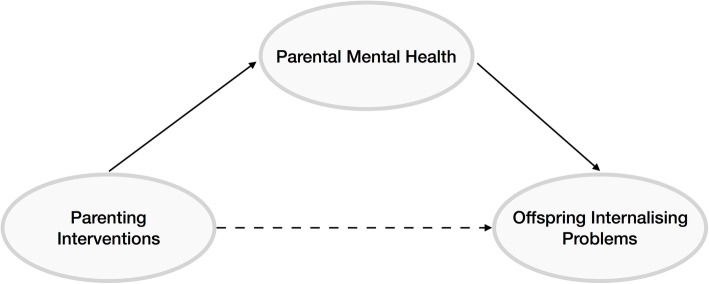
Parental mental health mediates the effect of parenting intervention on offspring internalising problems

**Fig. 3:**
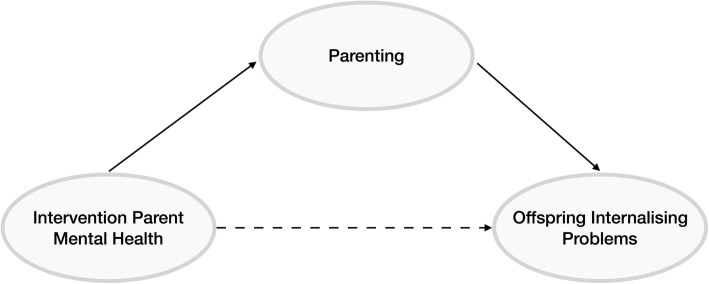
Treating parent mental health is a causal component of parenting interventions, parenting mediates the effect

As described above, parental mental health is important both for its influence on parenting practices and for its independent influence on child mental health. Therefore, understanding how parental mental health may influence the success of parenting interventions on child internalising outcomes is important. We propose three possibilities: firstly, parental mental health may moderate the effectiveness of interventions, whereby interventions may be more or less effective in parents with mental health problems (Fig. 1). In this case, parental mental health should be considered an effect modifier. Secondly, interventions may improve child mental health by improving parent mental health; in other words, parental mental health is a mediator of the associations (Fig. 2). When evaluating the effectiveness of parenting interventions, we will investigate as a secondary outcome the caregivers’ psychosocial well-being (e.g., anxiety, depression, self-esteem, sense of self-efficacy) [54]. Thirdly, treating parental mental health may be a causal component of parenting interventions, where dealing with mental health problems in the parent improves the parenting behaviour (even if it does not improve the parents’ mental health) (Fig. 3). In this case, interventions including a focus on parent mental health may be most effective.

Several recent reviews [54–59] have attempted to answer similar questions to ours in the present review, but with different populations or restricted interventions/outcomes. We briefly summarise their findings below.

Loechner et al. [55] conducted a meta-analysis of intervention studies (*N* = 14) designed to prevent the onset of depression symptoms and diagnoses in children and adolescents (under the age of 18) of depressed parents. The authors [55] reported positive but small effects on symptoms and diagnoses of depression in pooled estimates compared to no intervention. Only studies of parents who presented with depressive symptoms (selective prevention) were included [55], thereby excluding parents with other forms of psychopathology or parents without mental health problems (universal prevention). Other systematic reviews have limited their focus to interventions conducted in single countries and or to one type of intervention (e.g., Filene et al. [57] only included the US studies and home-visitation programmes), excluded internalising problems [56] or mental health problems in general [57] as outcomes, or focused exclusively on group-based rather than individual parenting interventions [52].

Importantly, parenting interventions have almost extensively been studied in the context of developing treatments for externalising disorders [60]. For example, in a meta-analysis by Kaminsky [56], the parenting training components Positive Interactions with Child, Time Out, Consistent Responding, and Practicing with Own Child were found to have positive effects on preventing or reducing children’s externalising problems. Overall, whilst there is a substantial variety of parenting interventions currently available [52, 54, 61–63], the evidence of their specific effectiveness in preventing child internalising disorders is mixed with some studies showing a positive effect, some showing no effect [52, 64].

### How the interventions might work—i.e., breaking down into causal components

Despite observational evidence of associations between different parenting practices (e.g., overprotective parenting, criticism) and later internalising problems in children [27], little empirical evidence exists to support the role of specific parenting intervention components in the onset, management and treatment of child internalising problems specifically [65, 66]. It is therefore also unclear which *aspects* of existing interventions may be most effective for preventing internalising problems. Thus, in addition to a lack of synthesised evidence for internalising problems specifically, there is also currently a limited understanding of the relative importance of the various *components* of parenting interventions which may be the *active* causal ingredients [67, 68]. Given the variety of existing interventions [69], their corresponding components, the number of possible combinations of intervention components and different comparisons across studies, it is currently unclear which components are driving any effectiveness of parenting interventions.

Currently, there is little understanding of how child internalising problems can be prevented through parenting programmes, as well as a lack of understanding of which specific components of these programmes may confer the strongest effects. A meta-analysis [57] of home visitation programmes did examine intervention components and found no consistent pattern (e.g., programme content, service delivery and research design) to be associated with a range of child and parent outcomes. However, two programme components resulted in better child cognitive outcomes: those which trained parents to respond sensitively and those which used parent role-play or other practising skills as part of the training [57]. Yap et al. [59] used a sub-group analysis to examine intervention components such as timing and focus (e.g., parent-child relationship, parenting skills, focus on parent mental health). Interestingly, they found that interventions that did include a focus on parental mental health were not effective for reducing internalising problems in children. This may highlight the need to focus on parenting. Kaminski et al. [56] meta-analysed components of 77 parent training interventions administered to parents of children ages 0–7 years. However, child internalising problems were not examined as an outcome. Thus, no meta-analysis to date has focused on components of parenting interventions in early childhood (ages 0–3) for reducing the risk for child internalising problems.

The current review aims to provide an up-to-date analysis of the effectiveness of parenting interventions for preventing child internalising problems. We will improve upon previous reviews by comparing parenting interventions not just with control groups but with other parenting interventions, even when they have not been directly compared in a trial, using network meta-analysis (NMA). In addition, we aim to break down intervention programmes into components of different aspects of parenting training (such as focus on cognitions, behaviour or emotions). This is because different components are hypothesised to differentially impact risk of offspring internalising problems based on observational epidemiological literature. If the network is connected at the component level, we will use a component NMA to assess the effectiveness of specific components (either individually or in combination). Finally, we will also improve upon prior work by including all interventions which aimed to improve parenting behaviours, but which may have not been specifically designed to prevent children’s internalising problems. We propose that there are likely common causal parenting mechanisms that could prevent both externalising and internalising problems (e.g., consistent and calm parenting). Therefore, interventions only focused on reducing externalising problems may also be relevant for preventing internalising problems since behavioural problems which are often noted and reported first may, for example, underlie anxiety and emotional problems.

The investigation of which components are driving the greatest effects has the potential to disentangle the utility and benefit of each component across interventions.

### Objectives

The primary objective of this review is to assess the effectiveness of parenting interventions examined in randomised controlled trials (RCTs) which took place prenatally or in children up to 3 years and 11 months of age, in the prevention of child internalising problems up to late adolescence, 18 years and 11 months. Additionally, this systematic review will determine the most effective components of parenting interventions in preventing child and adolescent internalising problems.

The results will provide a clearer understanding of not only whether parenting interventions are effective at preventing internalising problems in children and adolescents, but which intervention components are the causal ingredients of any observed effects.

## Methods

This protocol was developed according to the (Preferred Reporting Items for Systematic Review and Meta-Analysis Protocols) PRISMA-P 2015 checklist [70] and was registered with PROSPERO (number CRD42020172251).

### Criteria for considering studies for this review

#### Types of studies

Randomised controlled trials and quasi-randomised controlled trials will be included in this study either where the unit of randomisation is the individual (individual randomised controlled trial - IRCT) or where the unit of randomisation is a group/cluster (group randomised controlled trial - GRCT). Adjustment for clustering will be conducted where the authors did not do so. RCTs are studies in which participants were randomly allocated to an experimental condition (intervention) or a control group (no treatment, treatment as usual (TAU), placebo control group, other interventions). The process of adjustment for clustering may lead to a greater risk of selection bias. Cross-over trials are study designs where participants receive all treatments (usually active intervention and comparator) in a random sequence, thereby acting as their own control. Cross-over trials in psychological interventions are unlikely to include a “washout” period, and they often assume the form of waiting-list control designs, where an individual or a group is first assigned to a waiting list and then they receive the active intervention. Only the first period of cross-over trials (e.g., waiting list and active intervention) will be included in this study.

#### Setting

Study settings included will be medical (e.g., primary care), clinical, community, educational/school-based, home and online. Where possible, we will classify the type of setting of the intervention for each study.

#### Time frame

No limit will be applied with regard to publication year.

#### Types of participants

Primary caregivers, either male or female, of any age, who are adoptive, biological, foster, single, adolescent, homosexual, divorced and incarcerated will be included in this review. We will include studies with caregivers experiencing common mental health problems (see below for mental health disorders to be excluded). However, if we find significant difference between interventions used to target caregivers with mental health disorders and those offered universally, we will conduct separate/subgroup analyses according to caregiver mental health status.

Studies with the following participant characteristics will be excluded: relatives of the child who are not in the role of primary caregiver; parents or children with a major medical condition which may lead to a specialised type of intervention (e.g., pre-term conditions, gestational-diabetes, major parent or child disabilities, including intellectual disabilities); and parents with current confirmed (but not a history of) substance misuse, psychosis or who are perpetrators of maltreatment of the partner or child. Children who received a diagnosis within the autistic spectrum disorder (ASD) will not be excluded unless they meet some of the abovementioned exclusion criteria. When a range of child ages is included in a trial, interventions which took place prenatally or when at least 75% of the children were younger than 3 years and 11 months will be included, which will be calculated using the mean, standard deviation and *z*-scores, assuming normality of distribution. This age limit is because the developmental stage of the child will be key in determining the nature of the intervention, and thus, it may not be appropriate to pool intervention components of older and younger children.

Eligible interventions must have directly measured child internalising problems at the end of the trial, either from parent, teacher, child or clinician reports up to age 18 years and 11 months. Where baseline measures of internalising problems are reported, these will be extracted. We anticipate this being less common given our specifications on child age.

#### Types of outcome measures

##### Primary outcomes

The following primary outcomes will be extracted only when validated measures were used.

Child/adolescent primary outcomes

Child/adolescent internalising problems, for example anxiety and depression symptomatology and disorders, will be included. Studies will be screened and data will be extracted independently by two researchers (IC and EP). Studies using reports of child outcomes from children, parents, teachers and clinical/medical personnel will be included. When videotaped (e.g., incredible years - IY), data will be included when validated scales have been used. We will examine child/adolescent outcomes at whatever time they are available. Assessment points will be classified according to the time-point at which they were measured [52]:

1. Post-intervention (at conclusion of programme/intervention delivery)
2. Short-term follow-up (within 12 months post-intervention)
3. Medium-term follow-up (1 to 3 years post-intervention)
4. Long-term follow-up (3 or more years post-intervention).

*Child/adolescent primary outcome measures*

The following measures of (but not limited to) child/adolescent internalising problems will be included:

1. Any standardised diagnostic instrument which assesses children’s internalising symptoms (depressive, anxious symptoms) will be included. Accepted diagnostic criteria include but are not limited to the Diagnostic and Statistical Manual of Mental Disorders (DSM-5) [71], International Classification of Diseases (ICD-10) criteria [72], Diagnostic Classification of Mental Health and Developmental Disorders of Infancy and Early Childhood (DC:0–3) [73] or Diagnostic Classification of Mental Health and Developmental Disorders of Infancy and Early Childhood (DC:0-5) [1].
2. Validated questionnaires which assess internalising symptoms in children and adolescents (also at the level of sub-scales) will be included.

When multiple scales were used, we will apply the following decision rules:

1. Instruments with strongest construct and predictive validity (i.e., higher sensitivity/specificity against a gold standard of a child driven diagnostic instrument) will be prioritised.
2. Scales specifically targeting internalising problems compared to scales developed for other aims with internalising problems as a subscale will be favoured.
3. Scales developed for the age-range of the population will be favoured upon scaled developed for the general population.
4. The most used scale across studies will be favoured to reduce heterogeneity due to measurement error.
5. Given that we conceptualise internalising problems on a continuum, we will prioritise the total internalising score where available, rather than internalising subscales.

##### Secondary outcomes

Secondary outcome data will be extracted from studies identified as meeting abovementioned inclusion criteria.

*Primary caregiver secondary outcomes*

Secondary outcomes will not be used as inclusion criteria when primary outcomes were not assessed, as the focus of our review is not on externalising disorders or parenting outcomes. However, broader effectiveness of parenting interventions will be considered, where possible, especially for related outcomes which may influence the likelihood of programme success.

Secondary primary caregiver outcomes (process measures) will be:

1. Caregiver’s mental health;
2. Caregiver’s parenting self-efficacy;
3. Parent-child relationship and family relationship measures;

Caregiver’s secondary outcomes will be considered as potential mechanisms of change. Validated measures such as those listed above, but not limited to, will be used.

*Child secondary outcomes*

The following child secondary outcomes will be considered when studies used (but not limited to) instruments such as:

1. Child behavioural problems (e.g., conduct problems, aggressive behaviours, bullying, peer aggression) as measured by validated tools.
2. School achievement or attendance (e.g., years of education, drop-out).
3. Cognitive measures, where validated tools have been used.

In the current review, externalising problems will be considered only as secondary outcomes for three main reasons. First, as mentioned in the background, there is already a wealth of evidence available of parenting interventions aimed at the reduction of behavioural problems compared to the substantial lack of evidence for internalising problems. Second, externalising problems may underlie internalising symptoms; addressing the first without knowing whether the last were addressed could lead to an overestimation of positive effects of these interventions. Third, there is evidence of a specific increase in the past decades of internalising problems compared to behavioural problems [74]. This makes the question of this current review particularly important for both its novelty and potential utility.

*Adverse outcomes*

Adverse and negative outcomes will be considered. Child negative outcomes, parent negative outcomes (e.g., low self-esteem, depression, partner separation, family disruption) will be extracted when available. Attention to not only the possible positive effects but also the potential adverse intervention effects is important for a complete assessment of the effectiveness of an intervention [75].

Decision rules used for the primary outcomes will also be applied to secondary and adverse outcomes.

*Interventions*

In this review, parenting interventions are defined as those that have a central focus on parenting abilities, behaviours and beliefs. Specifically, the intervention should include active training in parenting abilities with or without other foci. They should be somewhat standardised (e.g., based on a structured manual, booklets or guidelines) in order to ensure reproducibility of the delivery by the staff to the parents [76]. Some of the most common parenting interventions include Family Cognitive Behavioural Therapy, Incredible Years (IY), Triple P, Mellow Parenting, Strengthening Families Strengthening Communities, and the Family Links Nurturing Programme (FLNP) [77, 78]. Specific parenting abilities targeted are expected to be wide and can include attention to nurturing skills, teaching abilities, discipline, monitoring, management, language, parent-child relationship, self-regulatory strategies and others (for a complete list see: “Parenting Matters: Supporting Parents of Children Ages 0-8” [69]). We will therefore exclude any individual medical, psychiatric and psychological therapy administered to the caregiver which does not specifically intervene on parental abilities or behaviours. Interventions which were delivered during pregnancy, post-partum or before the 4th birthday of the child will be included. Treatments that occurred preconception will not be considered in this systematic review. No limitations on the intensity (number and length of sessions) and length of follow-up will be imposed. Parenting programmes will be considered eligible regardless of the theoretical framework.

Given the complexity of parenting interventions, we aim to disaggregate interventions into key components using a component-based NMA [79]. Possible components could include different intervention foci, such as the specific behaviours, skills, emotions or cognitions the intervention targets. This approach will enable us to determine which intervention components (or grouped intervention components) are driving any treatment effect. Where intervention components are not reported, authors will be contacted, and missing information will be requested.

*Comparison*

All control group types will be included in this systematic review regardless of whether they received or were exposed to any other type of control intervention, or lack thereof. Eligible comparators will include wait list, TAU (treatment as usual), other treatment and no treatment. Other treatments may include information about parenting and infant/child development, information on the management of behavioural/emotional problems or other forms of psycho-/health education.

*Years of publications considered*

No limitation on the year of publication will be imposed.

*Language*

No language limitation will be imposed. In the event that an eligible study has been written in a language other than English, where possible, professional translators will be hired.

*Publication status*

Published and unpublished trials will be included in the review. Ongoing studies will be searched in the randomised controlled trial register (see website: https://clinicaltrials.gov/) and considered where relevant.

### Information sources

#### Search strategy

The search strategy was developed in collaboration with systematic review experts and with a medical librarian with expertise in systematic reviews. The search strategy includes MESH Terms and keywords and search strategy terms which were harmonised for each database (search of MEDLINE is reported below). Existing systematic reviews and meta-analyses known to the authors will also be hand-searched. The search will be re-run prior to the final analysis.

#### OVID, MEDLINE from 1946 to present

Exp *Caregivers/ or exp *parents/ or exp *Legal Guardians/ or caretaker.mp. or custodian.mp. or exp *Pregnancy/ or exp *maternal behavior/ or exp *parent-child relations/ or exp *parenting/ or exp *paternal behavior/ or expectan*.mp. or exp *postnatal/ or exp *post-natal/ or exp *post-partum/ or exp *perinatal/ or exp *prenatal/ or exp *antenatal/ or parent-child relations/ or exp *father-child relations/ or exp *mother-child relations/ or exp *parenting/

AND

exp *preventive health services/ or exp *Prenatal Education/ or exp *Perinatal Care/ or exp *"early intervention (education)"/ or exp *early medical intervention/ or *primary prevention/ or *secondary prevention/ or exp *tertiary prevention/ or exp *Psychotherapy/ or program*.mp. or coach*.mp. or training*.mp.

AND

exp *control groups/ or exp *cross-over studies/ or exp *double-blind method/ or exp *random allocation/ or exp *single-blind method/ or randomi#ed controlled trial.mp. or exp *clinical trials as topic/ or exp *controlled clinical trials as topic/ or exp *randomized controlled trials as topic/

#### Filters

No language or date of publication filter will be applied. Publication type filter will be applied in each database where possible.

#### Electronic sources

The following electronic databases will be searched:

- Cochrane Central Register of Controlled Trials (CENTRAL) (1996-present)
- Ovid Medical Literature Analysis and Retrieval System Online (MEDLINE) (1949-present)
- Ovid Excerpta Medica Database (EMBASE) (1974-present)
- Ovid PsycINFO (1806-present)
- Education Resources Information Center (ERIC) (1966-present)
- ClinicalTrials.gov (1997-present)

RCTs registered at https://clinicaltrials.gov/ but not yet published will be screened and included when eligible.

#### Searching other resources

##### Reference lists

Reference lists of relevant systematic reviews and individual studies will be hand-searched.

Grey literature will be searched and known experts in the field will be contacted to explore the possibility of any unpublished research. Where critical information is not reported in published research, study authors will be contacted.

##### Study records

Deduplication will be carried out using Endnote

##### Data management

Rayyan [80] software will be used for data management and study screening.

##### Selection process

**Fig. 4:**
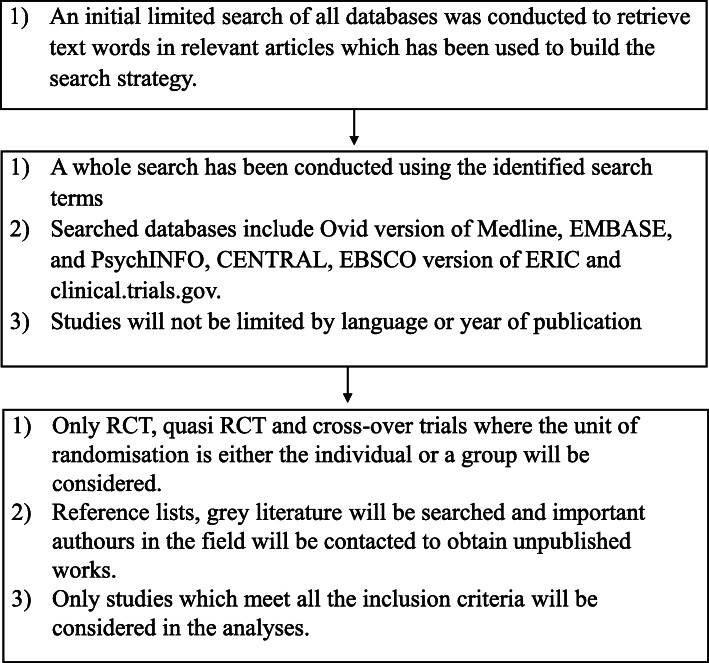
Flow chart of selection process

Following the deduplication process in Endnote, the selection process will be conducted independently by two review authors (IC and EP), who will screen and identify studies based on inclusion and exclusion criteria (Fig. 4, Table 1).

**Table 1:** Inclusion and exclusion criteria

Inclusion criteria	Exclusion criteria
**Population**	
Primary caregivers of infants and toddlers up to 3 years and 11 months.	Studies including specific groups of caregivers with intellectual disabilities and with current mental health problems such as schizophrenia, substance misuse and abuse, and children born preterm, at low birth weight or with congenital diseases.
**Intervention**	
Structured psychosocial parenting intervention delivered either antenatal or within the child’s first 3 years and 11 months of life.	Interventions not focusing specifically on parenting, interventions delivered at preconception or unstructured interventions.
**Comparator**	
No restrictions will be imposed.	
**Outcome**	
Child and/or adolescent internalising problems up to age 18 and 11 months.	Studies reporting only externalising problems or cognitive or health related outcomes.
**Design**	
Randomised controlled trials (RCT) or quasi-RCTs either with individual or group levels of randomization. Cross-over trials.	Study designs such as quasi experimental studies (e.g., pre-post test), case control, cohort, cross-sectional and systematic reviews
**Publication type**	
No restrictions will be imposed.	

The first selection of potentially eligible studies will be conducted by screening titles and abstracts in Rayyan. Disagreements between reviewers will be resolved through discussion and consensus. Where consensus is not possible, a third external reviewer (RMP) will resolve any disagreements. After the first round of screening is completed, the full texts of the resulting studies will be screened for eligibility. These articles will in turn be further screened independently by the two reviewers (IC and EP) according to the process outlined above. Detailed reasons for exclusion will be tracked and reported in the PRISMA flow diagram. Multiple reports of the same studies will be considered together. Included articles will receive an identification number before data extraction.

#### Data extraction and management

Data from included studies will be extracted and risk of bias assessments will be performed independently by two reviewers (IC and EP). Data regarding study design, intervention characteristics (type of intervention, theoretical framework of the intervention, experience of the provider, length, intensity, outcomes), participant characteristics, comparators, delivery mode, setting, attrition rates, outcome measures and effect sizes will be extracted and entered into an eletronic tool such as RevMan or Systematic Review Data Repository (SRDR) [81, 82].

Unadjusted results will be preferred over adjusted results to improve consistency across studies and reduce the potential for selective reporting bias. If missing data cannot be obtained, the Cochrane Practical Methods for Handling Missing Data will be used [83, 84].

#### Data items

##### Study design

RCTs, quasi-randomised trials and cross-over trials at the individual and group level will be eligible for inclusion. Information relevant for assessing risk of bias will be extracted using the Cochrane tool [85] (e.g., allocation method, randomisation).

##### Participants

Participant characteristics (of the caregiver and the child) that may modify treatment effects will be extracted and reported. These will include baseline psychopathology, gender, age, comorbidity status, presence and numerosity of previous mental health-related conditions, and psychiatric medication use. The number of participants at baseline and those lost to drop-out will also be extracted.

##### Intervention

Data on other potential intervention modifiers such as treatment length, intensity (frequency of sessions), length of follow-up, expertise of the therapist and measures used will be included.

##### Comparator

Data on the type of comparator used as well as data regarding control group participants will be extracted.

#### Risk of bias in individual studies

The risk of bias assessment will be conducted independently by two reviewers (IC and EP) using the Cochrane Collaboration’s Risk of Bias Assessment Tool [85]. In cases of disagreement, consensus will be reached through discussion and where not possible to obtain consensus, a third reviewer (RMP) will be included in the process.

The Cochrane Risk of Bias Assessment Tool [85] assesses the following potential sources of bias:

1. Random sequence generation
2. Allocation concealment
3. Blinding of participants and personnel
4. Blinding of outcome assessment
5. Incomplete outcome data
6. Selective reporting
7. Other sources of bias

Each category will be scored as at low, high or unclear risk of bias.

#### Sequence generation

Allocation methods will be assessed to determine the potential of bias due to the creation of incomparable groups.

#### Allocation concealment

Adequacy of concealment in the participant allocation process will be assessed. Randomised and quasi-randomised methods of concealment will be considered good enough. Potential for bias due to inadequate concealment of the allocation process will also be assessed.

#### Blinding

Due to the nature of the interventions, double blinding is not possible. We will therefore evaluate whether personnel were blinded when allocating participants to the different conditions and whether outcome assessors were blinded as to which intervention group participants were in.

#### Incomplete outcome data

The intention-to-treat (ITT) approach is widely used in the estimation of treatment effects from RCTs because of its low risk for bias [86]. Where studies did not report an intention-to-treat analysis, authors will be contacted in an attempt to obtain missing data. How the authors dealt with incomplete data and how data on attrition and exclusion were reported will also be evaluated. Imputation methods for handling missing data are described below.

#### Selective outcome reporting

Any evidence of attempts by the authors to omit the reporting of relevant outcomes will also be assessed.

#### Measures of treatment effect

##### Dichotomous outcome data

For dichotomous outcomes, intervention effectiveness will be summarised as odds ratios (OR) or risk ratios (RR) and presented with 95% confidence intervals (CIs) and standard deviations (SDs). We will utilise the Number Needed to Treat (NNT) [68, 84] approach to determine how many participants are needed in the intervention group to observe the expected outcome.

##### Continuous outcome data

For continuous outcomes, data from treatment completers will be pooled by calculating the mean differences (MDs) between groups. If trials measured the same outcome using different scales, standardised mean differences (SMDs), also known as Cohen’s *d*, will be estimated and reported with 95% CIs. The SMD is obtained by subtracting the mean obtained in the control group from the mean obtained in the intervention group and dividing this value by the standard deviation of the outcome among participants. Where it is not possible to calculate SMDs, *t* tests, *F* tests, *χ*^2^, *p* values, eta-squared and beta coefficients will be used and reported. When SMDs differ from zero (based on 95% CIs), treatment and control groups will not be considered equivalent. If improvement is associated with lower scores on the outcome measure (e.g., fewer anxious or depressive symptoms), negative SMD values will be interpreted as the treatment being more effective than the control and vice versa [87]. Together with CIs, SMDs will be interpreted as follows: small effect size, SMD = 0.2; medium effect size, SMD = 0.5; and large effect size, SMD = 0.8 [88]. When baseline information is available, we will compare pre- and post-test measures as a standardised mean change as indicated in literature [89, 90].

#### Unit of analysis issues

##### Cluster-randomised trials

If studies have not taken clustering into account, for example, where only raw or observed means and SDs are reported, methods in Section 16.3.4 of the Cochrane Handbook [91] will be used to perform approximately correct analyses. Data from cluster randomised trials will only be included in meta-analyses if clustering has been quantified and reported using an intra-cluster-correlation coefficient (ICC), or if other approximately correct analyses can be performed.

##### Repeated observations on participants

Studies reporting long-term outcomes will be included. Since the combination of outcomes with variable length of follow-up can lead to unit-of-analysis error in standard meta-analysis, separate analyses based on previously defined length of follow-up [92] will be carried out.

##### Dealing with missing data

In the case of missingness of relevant data, authors will be contacted to request sufficient information to conduct an intention-to-treat analysis. Where possible we will describe participant characteristics for whom data are missing and we will analyse the proportion of missingness as a function of the number of participants included in the analyses and total number of participants in the study (e.g., how many people were initially included in the study and for how many people outcome data are available). We will employ different methods to handle missing data according to the nature of missingness. Missing data due to dropout/attrition will be included in the risk of bias assessment.

##### Dichotomous outcome data

Missing dichotomous data will be assumed to be missing not at random (MNAR) or informatively missing (IM) [93]. This approach assumes participants have dropped-out for some reason (for example participant’s mental health). We will assume that participants who dropped-out after the allocation process did so for negative reasons, for example non-response to the intervention (e.g., the intervention did not lead to an improvement).

A recommended simple imputation for dichotomous missing data is the best-case and worst-case scenario [94, 95]. In the best-case scenario, the assumption will be that all missing participants dropped-out because of a positive outcome in the experimental group and a negative or null outcome in the control group. Conversely, in the worst-case scenario, participants dropped out because the treatment had a negative or null effect, whilst the control group led to a positive outcome. In the case of a large amount of missing data, results obtained may be unrealistic because they will reflect two extreme scenarios [86, 96].

##### Continuous outcome data

When continuous outcome data are missing or outcomes were not recorded at time-point of interest, the Last Observation Carried Forward (LOCF) imputation method (using the last observed non-missing values) [97] will be used to fill-in missing values, whenever these data are available. Imputation methods will be considered carefully because of their potential to lead to biased results (e.g., overestimating or underestimating the effectiveness of an intervention).

Missing standard deviations (SDs) will be calculated from *p* values, *t* test statistics, confidence intervals (CIs) and standard errors (SEs).

##### Assessment of heterogeneity

*Data synthesis*

Clinical and methodological heterogeneity will be assessed by examining variation across studies in participant characteristics, intervention type and delivery mode, outcomes or other relevant study characteristics such as concealment method or blinding procedures.

*Network meta-analysis*

**Fig. 5:**
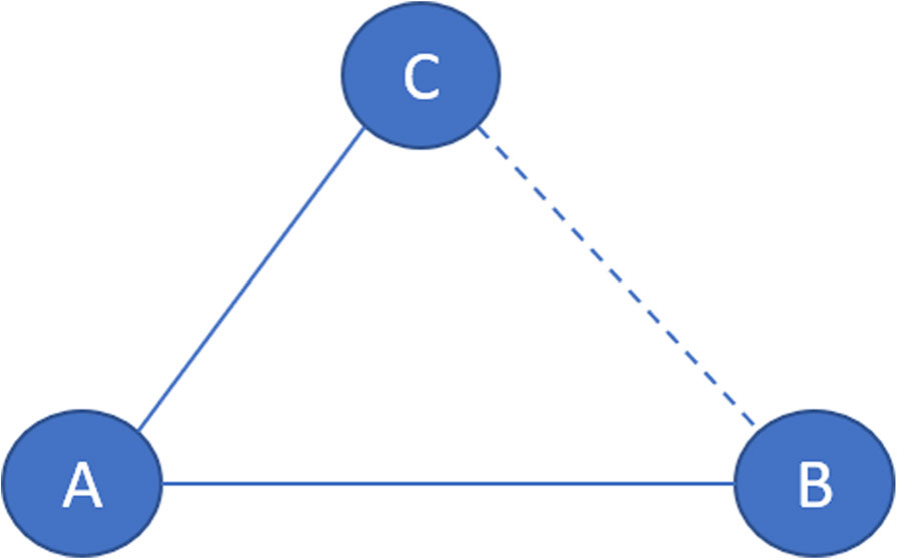
Illustration of indirect treatment comparison in an NMA

Results will be analysed using a network meta-analysis (NMA) [98]. An NMA utilises a connected network of interventions. NMA allows to assess the comparative effectiveness of several competing interventions for a condition, as long as all the trials included in the analysis form a connected network [67, 68, 99]. The idea behind NMA is a simple one: when head-to-head evidence comparing interventions B and C is not available, evidence on the BC intervention effect can be obtained indirectly via trials comparing AB and AC (Fig. 5). This enables all pairwise effects to be estimated indirectly, even in the absence of direct evidence, whilst respecting the randomised structure of the evidence [100].

In the first instance, we will fit a model which compares interventions as ‘clinically meaningful units’, i.e., at the whole intervention level. If appropriate, we will also conduct a component-level NMA, where the ‘active ingredients’ of interventions are modelled using a network meta-regression approach [79]. We will explore component effects using an additive main effects model, as well as a full interaction (multiplicative) model where each unique combination of components is regarded as a separate intervention.

##### Data analysis

All statistical analyses will be conducted in a Bayesian framework using OpenBUGS software (www.openbugs.net). OpenBUGS is commonly used software for conducting NMA in a Bayesian framework due to its flexibility and availability of programme code [101].

Statistical heterogeneity is anticipated given potential variation in participant characteristics, intervention type and mode of delivery, and outcome measures. Random effects models, assuming a common between-study variability, will be used to address resulting statistical heterogeneity. The goodness of fit of each model to the data will be assessed using the posterior mean residual deviance, defined as the difference between the deviance for the fitted model and the saturated model, where deviance quantifies model fit using the likelihood function. Models will be compared using the Deviance Information Criterion (DIC), calculated by summing the posterior mean of the residual deviance and the effective number of parameters. The DIC penalises the posterior mean residual deviance (model fit) by the effective number of parameters in the model (model complexity) and therefore takes both model fit and complexity into account.

NMA validity depends on the consistency assumption. That is, that there is no intervention effect modification by treatment comparison or that the frequency of effect modifiers is similar across the included studies. This assumption can be examined by assessing the inclusion/exclusion criteria of every intervention in the network to determine whether participants, intervention protocols and administration, etc., are similar in ways that may modify treatment effects. Trial and participant characteristics (such as maternal depression) will therefore be compiled into a table to facilitate and visually inspect the ‘similarity’ of factors we consider likely to modify treatment effects.

##### Assessment of reporting biases

Potential small-study effects will be examined by including study size as a covariate in meta-regression analyses. Funnel plot asymmetry will be tested [102] to explore potential reporting bias (i.e., publication bias, selective outcomes reporting and selective analysis reporting). In addition, we will try to reduce selective outcomes reporting bias by not excluding studies based on the outcomes during the first screening, as recommended in Cochrane Handbook for Systematic Reviews of Interventions, Sections 8.7 and 7 [103, 104], and by checking, where possible, for discrepancies between outcomes reported in protocols and outcomes reported in the published articles.

##### Subgroup analysis and investigation of heterogeneity

We anticipate that where possible, we will conduct subgroup analyses on parent characteristics (e.g., gender, age, previous or present psychopathology); child characteristics (e.g., gender, age, comorbidities); socio-economic status of the household and of the broader environment (e.g., household income, low vs high income country); programme administrator characteristics (e.g., years of expertise), intervention features (e.g., comparator, intensity or length of the intervention, theoretical framework); and outcome characteristics (e.g., short vs long-term follow-up) [105]. We will attempt to include all possible relevant modifying factors and exclude prognostic factors. Unfortunately, especially in psychological research, these factors (modifiers and prognostic factors) often overlap and their potential role is difficult to disentangle, particularly at the protocol stage. Finally, individual’s genetic liability to mental health problems may represent an important modifier and/or prognostic factor. Whilst genetic risk is not modifiable (i.e., it does not represent a viable target of interventions) and it is unlikely that it is measured in the studies eligible for this review, the use of a well-performed random or quasi-random allocation should minimise imbalances in important prognostic variables or effect modifiers across intervention and control arms, allowing inferences to be made on the effectiveness of parenting interventions on child and adolescent internalising problems.

Once we have obtained the studies, we will examine the presence of potential modifiers and prognostic factors, and which modifiers have the scientific dignity to be included as post hoc analyses, as recommended in Cochrane Handbook for Systematic Reviews of Interventions, Section 9.6.5 [91].

In the selection of characteristics to be included in our meta-analysis, we will consider that certain relationships may be confounded. For example, we may not find any effect of the intensity of an intervention because it is closely related to the severity of the condition of the participants, which could bias our findings.

##### Sensitivity analyses

Sensitivity analyses will be conducted by excluding studies at high or unclear risk of bias on allocation concealment and blinding domains per the Cochrane Risk of Bias Assessment Tool [85]. Sensitivity analyses will include the following:

1. Fixed-effect analyses for the pairwise and network meta-analyses;
2. Where missing data were imputed, trials will be removed where exchangeability assumptions were not met;
3. Trials that used a non-operationalised diagnostic criteria will be removed.

##### Confidence in cumulative evidence

We will use an appropriate tool to summarise and assess the quality of the evidence across included studies [106]. This might include Grades of Recommendations, Assessment, Development, and Evaluation (GRADE) [107], Confidence in Network Meta-Analysis (CINeMA) [108] or threshold analysis [109].

## Discussion

The proposed systematic review and meta-analysis aims to address questions about the effectiveness of parenting interventions in the prevention of child and adolescent internalising problems. These questions are important for various reasons. As mentioned in the introduction, internalising problems are common and they represent an important economical and societal burden. In addition, the age of onset of anxiety and mood disorders differs, with anxiety disorders starting at earlier ages and mood disorders being more prevalent in late adolescence/young adulthood, with evidence that the peak acceleration in depressed mood is age 13 for females and age 16 for males [13, 110, 111]. Further, internalising problems often present as comorbid with other disorders in children and adolescents [112, 113], and often precede other psychiatric disorders [12, 114, 115]. The quality of parent-child relationships in early childhood represents a unique time window for child emotional and social development [44], providing a potentially salient period for prevention and intervention. Developing effective preventive interventions could help reduce the societal and economic burden of mental health problems, both by reducing the onset of new cases (incidence) or by attenuating symptoms in individuals already affected.

The findings of this review and meta-analysis have the potential to inform NICE Public Health guidance.

Possible limitations of this review include the exclusion of interventions aimed at secondary caregivers due to the reduction in power introduced by subgroup analysis, potential difficulties in disentangling moderators from prognostic factors in subgroups analyses and the possible role of confounding factors in biasing finding estimates.

### Starting date

The search strategy was run on the 8^th^ of March 2020. In April 14, we are currently screening by title and abstract.

## Data Availability

Not applicable

## Abbreviations

NMA: Network meta-analysis
RCT: Randomised controlled trial
DALY: Disability-adjusted life-year
PRISMA-P: Preferred Reporting Items for Systematic Review and Meta-Analysis Protocols
PROSPERO: International Prospective Register of Systematic Reviews
IRCT: Individual randomised controlled trial
GRCT: Group randomised controlled trial
TAU: Treatment as usual
DSM-5: Diagnostic and Statistical Manual of Mental Disorders
ICD-10: International Classification of Diseases
ASD: Autistic spectrum disorder
IY: Incredible years
FLNP: Family Links Nurturing Programme
MESH: Medical Subject Headings
DC:0-3: Diagnostic Classification of Mental Health and Developmental Disorders of Infancy and Early Childhood
DC:0-5: Diagnostic Classification of Mental Health and Developmental Disorders of Infancy and Early Childhood
CENTRAL: Cochrane Central Register of Controlled Trials
MEDLINE: Medical Literature Analysis and Retrieval System Online
EMBASE: Excerpta Medica Database
ERIC: Education Resources Information Center
ITT: Intention-to-treat
OR: Odds ratios
RR: Risk ratios
CIs: Confidence intervals
SDs: Standard deviations
NNT: Number needed to treat
MDs: Mean differences
SMDs: Standardised mean differences
ICC: Intra-cluster-correlation coefficient
MNAR: Missing not at random
IM: Informatively missing
LOCF: Last observation carried forward
SEs: Standard errors
DIC: Deviance information criterion
GRADE: Grades of Recommendations, Assessment, Development, and Evaluation
CINeMA: Confidence in Network Meta-Analysis

## References

[1] Zeanah CH, Carter AS, Cohen J, Egger H, Gleason MM, Keren M (2016). Diagnostic classification of mental health and developmental disorders of infancy and early childhood dc: 0–5: selective reviews from a new nosology for early childhood psychopathology. Infant Ment Health J.

[2] Egger HL, Angold A. Common emotional and behavioral disorders in preschool children: presentation, nosology, and epidemiology. J Child Psychol Psychiatry [Internet]. 1;47(3-4):313–37Available from; 2006. https://doi.org/10.1111/j.1469-7610.2006.01618.x, https://doi.org/10.1111/j.1469-7610.2006.01618.x. 10.1111/j.1469-7610.2006.01618.x 16492262

[3] Egger HL, Angold A (2009). Classification of psychopathology in early childhood. Handbook of infant mental health, 3rd ed.

[4] Brotman MA, Schmajuk M, Rich BA, Dickstein DP, Guyer AE, Costello EJ (2006). Prevalence, clinical correlates, and longitudinal course of severe mood dysregulation in children. Biol Psychiatry [Internet].

[5] Wichstrøm L, Berg-Nielsen TS, Angold A, Egger HL, Solheim E, Sveen TH. Prevalence of psychiatric disorders in preschoolers. J Child Psychol Psychiatry [Internet]. 2012 1;53(6):695–705. Available from: https://doi.org/10.1111/j.1469-7610.2011.02514.x. 10.1111/j.1469-7610.2011.02514.x 22211517

[6] Baranne ML, Falissard B. Global burden of mental disorders among children aged 5–14 years. Child Adolesc Psychiatry Ment health [Internet]. 2018;12(1):19. Available from: https://doi.org/10.1186/s13034-018-0225-4. 10.1186/s13034-018-0225-4 PMC5896103 29682005

[7] Whiteford HA, Degenhardt L, Rehm J, Baxter AJ, Ferrari AJ, Erskine HE (2013). Global burden of disease attributable to mental and substance use disorders: findings from the global burden of disease study 2010. Lancet [Internet].

[8] Kessler RC, Aguilar-Gaxiola S, Alonso J, Chatterji S, Lee S, Ormel J, et al. The global burden of mental disorders: an update from the WHO World Mental Health (WMH) Surveys. Epidemiol Psichiatr Soc [Internet]. 2011/04/11. 2009;18(1):23–33. Available from: https://www.cambridge.org/core/article/global-burden-of-mental-disorders-an-update-from-the-who-world-mental-health-wmh-surveys/FCE4DE10C9FB1A9FFDDDBA3C5264F445. 10.1017/s1121189x00001421 PMC3039289 19378696

[9] Klein AM, Schlesier-Michel A, Otto Y, White LO, Andreas A, Sierau S, et al. Latent trajectories of internalizing symptoms from preschool to school age: A multi-informant study in a high-risk sample. Dev Psychopathol [Internet]. 2018/04/29. 2019;31(2):657–81. Available from: https://www.cambridge.org/core/article/latent-trajectories-of-internalizing-symptoms-from-preschool-to-school-age-a-multiinformant-study-in-a-highrisk-sample/B5BFE47419F6F418579C876A2FF05FF6. 10.1017/S0954579418000214 29704908

[10] Melkevik O, Nilsen W, Evensen M, Reneflot A, Mykletun A (2016). Internalizing disorders as risk factors for early school leaving: a systematic review. Adolesc Res Rev.

[11] LIU J, CHEN X, LEWIS G (2011). Childhood internalizing behaviour: analysis and implications. J Psychiatr Ment Health Nurs.

[12] Lancefield KS, Raudino A, Downs JM, Laurens KR. Trajectories of childhood internalizing and externalizing psychopathology and psychotic-like experiences in adolescence: a prospective population-based cohort study. Dev Psychopathol [Internet]. 2016/02/09. 2016;28(2):527–36. Available from: https://www.cambridge.org/core/article/trajectories-of-childhood-internalizing-and-externalizing-psychopathology-and-psychoticlike-experiences-in-adolescence-a-prospective-populationbased-cohort-study/71A58C5E5F0E16DA3159355F851C4F63. 10.1017/S0954579415001108 PMC4855987 26856897

[13] Kessler RC, Berglund P, Demler O, Jin R, Merikangas KR, Walters EE. Lifetime prevalence and age-of-onset distributions of DSM-IV disorders in the national comorbidity survey replication. Arch Gen Psychiatry [Internet]. 2005 Jun 1;62(6):593–602. Available from: https://doi.org/10.1001/archpsyc.62.6.593. 10.1001/archpsyc.62.6.593 15939837

[14] Lyons-Ruth K, Todd Manly J, Von Klitzing K, Tamminen T, Emde R, Fitzgerald H, et al. The worldwide burden of infant mental and emotional disorder: report of the task force of the world association for infant mental health. Infant Ment Health J [Internet]. 2017 1;38(6):695–705. Available from: https://doi.org/10.1002/imhj.21674. 10.1002/imhj.21674 29088514

[15] von Klitzing K, Döhnert M, Kroll M, Grube M. Mental disorders in early childhood. Dtsch Arztebl Int. 2015/05/25. 2015 May;112(21–22):375–86. 10.3238/arztebl.2015.0375 PMC4496484 26149380

[16] Franz L, Angold A, Copeland W, Costello EJ, Towe-Goodman N, Egger H (2013). Preschool anxiety disorders in pediatric primary care: prevalence and comorbidity. J Am Acad Child Adolesc Psychiatry.

[17] Keenan K, Shaw DS, Walsh B, Delliquadri E, Giovannelli J (1997). DSM-iii-r disorders in preschool children from low-income FAMILIES. J Am Acad Child Adolesc Psychiatry [Internet].

[18] Polderman TJC, Benyamin B, De Leeuw CA, Sullivan PF, Van Bochoven A, Visscher PM (2015). Meta-analysis of the heritability of human traits based on fifty years of twin studies. Nat Genet.

[19] Boomsma DI, Van Beijsterveldt CEM, Hudziak JJ. Genetic and environmental influences on anxious/depression during childhood: a study from the Netherlands Twin Register. Genes, Brain Behav [Internet]. 2005 1;4(8):466–81. Available from: https://doi.org/10.1111/j.1601-183X.2005.00141.x. 10.1111/j.1601-183X.2005.00141.x 16268991

[20] Demirkan A, Penninx BWJH, Hek K, Wray NR, Amin N, Aulchenko YS (2011). Genetic risk profiles for depression and anxiety in adult and elderly cohorts. Mol Psychiatry.

[21] Sullivan PF, Neale MC, Kendler KS (2000). Genetic epidemiology of major depression: review and meta-analysis. Am J Psychiatry.

[22] Wray NR, Pergadia ML, Blackwood DHR, Penninx B, Gordon SD, Nyholt DR (2012). Genome-wide association study of major depressive disorder: new results, meta-analysis, and lessons learned. Mol Psychiatry.

[23] Eley TC, Bolton D, O’Connor TG, Perrin S, Smith P, Plomin R (2003). A twin study of anxiety-related behaviours in pre-school children. J Child Psychol Psychiatry.

[24] Hawes DJ, Allen J. Evidence-based parenting interventions. in: positive mental health, fighting stigma and promoting resiliency for children and adolescents. Elsevier; 2016. p. 185–204.

[25] Stein A, Pearson RM, Goodman SH, Rapa E, Rahman A, McCallum M (2014). Effects of perinatal mental disorders on the fetus and child. Lancet..

[26] Yap MBH, Pilkington PD, Ryan SM, Jorm AF. Parental factors associated with depression and anxiety in young people: a systematic review and meta-analysis. J Affect Disord [Internet]. 2014;156:8–23. Available from: http://www.sciencedirect.com/science/article/pii/S0165032713008057. 10.1016/j.jad.2013.11.007 24308895

[27] Rose J, Roman N, Mwaba K, Ismail K. The relationship between parenting and internalizing behaviours of children: a systematic review. Early Child Dev Care [Internet]. 2018 3;188(10):1468–86. Available from: https://doi.org/10.1080/03004430.2016.1269762.

[28] Ashford J, Smit F, Van Lier PAC, Cuijpers P, Koot HM. Early risk indicators of internalizing problems in late childhood: a 9-year longitudinal study. J Child Psychol Psychiatry [Internet]. 2008 1;49(7):774–780. Available from: https://doi.org/10.1111/j.1469-7610.2008.01889.x. 10.1111/j.1469-7610.2008.01889.x 18341546

[29] Rochat TJ, Houle B, Stein A, Pearson RM, Bland RM. Prevalence and risk factors for child mental disorders in a population-based cohort of HIV-exposed and unexposed African children aged 7–11 years. Eur Child Adolesc Psychiatry [Internet]. 2018;27(12):1607–1620. Available from: https://doi.org/10.1007/s00787-018-1146-8. 10.1007/s00787-018-1146-8 29680970

[30] Kovacs M, Lopez-Duran N (2010). Prodromal symptoms and atypical affectivity as predictors of major depression in juveniles: implications for prevention. J Child Psychol Psychiatry.

[31] Hopkins J, Lavigne JV, Gouze KR, LeBailly SA, Bryant FB (2013). Multi-domain models of risk factors for depression and anxiety symptoms in preschoolers: evidence for common and specific factors. J Abnorm Child Psychol.

[32] Bennett DS, Sullivan MW, Lewis M (2010). Neglected children, shame-proneness, and depressive symptoms. Child Maltreat.

[33] Paulus FW, Backes A, Sander CS, Weber M, von Gontard A (2015). Anxiety disorders and behavioral inhibition in preschool children: a population-based study. Child Psychiatry Hum Dev.

[34] Dougherty LR, Tolep MR, Bufferd SJ, Olino TM, Dyson M, Traditi J (2013). Preschool anxiety disorders: comprehensive assessment of clinical, demographic, temperamental, familial, and life stress correlates. J Clin Child Adolesc Psychol.

[35] Hudson JL, Dodd HF, Lyneham HJ, Bovopoulous N (2011). Temperament and family environment in the development of anxiety disorder: two-year follow-up. J Am Acad Child Adolesc Psychiatry.

[36] Edwards SL, Rapee RM, Kennedy S (2010). Prediction of anxiety symptoms in preschool-aged children: examination of maternal and paternal perspectives. J Child Psychol Psychiatry.

[37] Braungart-Rieker JM, Hill-Soderlund AL, Karrass J (2010). Fear and anger reactivity trajectories from 4 to 16 months: the roles of temperament, regulation, and maternal sensitivity. Dev Psychol.

[38] Lewis-Morrarty E, Degnan KA, Chronis-Tuscano A, Rubin KH, Cheah CSL, Pine DS (2012). Maternal over-control moderates the association between early childhood behavioral inhibition and adolescent social anxiety symptoms. J Abnorm Child Psychol.

[39] Griffin KW, Botvin GJ, Scheier LM, Diaz T, Miller NL (2000). Parenting practices as predictors of substance use, delinquency, and aggression among urban minority youth: moderating effects of family structure and gender. Psychol Addict Behav.

[40] Baumrind D, Moselle KA (1985). A developmental perspective on adolescent drug abuse. Adv Alcohol Subst Abuse.

[41] Cohen DA, Richardson J, LaBree L (1994). Parenting behaviors and the onset of smoking and alcohol use: a longitudinal study. Pediatrics..

[42] O’Donnell KJ, Meaney MJ. Fetal origins of mental health: the developmental origins of health and disease hypothesis. Am J Psychiatry [Internet]. 2016 14;174(4):319–328. Available from: https://doi.org/10.1176/appi.ajp.2016.16020138. 10.1176/appi.ajp.2016.16020138 27838934

[43] Jones PB, Rantakallio P, Hartikainen A-L, Isohanni M, Sipila P. Schizophrenia as a long-term outcome of pregnancy, delivery, and perinatal complications: a 28-year follow-up of the 1966 North Finland general population birth cohort. Am J Psychiatry [Internet]. 1998 1;155(3):355–364. Available from: https://doi.org/10.1176/ajp.155.3.355. 10.1176/ajp.155.3.355 9501745

[44] Bowlby J. Attachment and loss: volume I: attachment. In: Attachment and Loss: Volume I: Attachment. London: The Hogarth Press and the Institute of Psycho-Analysis; 1969. p. 1–401.

[45] Fox SE, Levitt P, Nelson CA III. How the timing and quality of early experiences influence the development of brain architecture. Child Dev [Internet]. 2010 1;81(1):28–40. Available from: https://doi.org/10.1111/j.1467-8624.2009.01380.x. 10.1111/j.1467-8624.2009.01380.x PMC2846084 20331653

[46] Schore AN. Affect regulation and the origin of the self: the neurobiology of emotional development. Routledge; 2015.

[47] Schore AN (2009). Attachment trauma and the developing right brain: origins of pathological dissociation. Dissociation and the dissociative disorders: DSM-V and beyond.

[48] Schore AN (2010). Relational trauma and the developing right brain: the neurobiology of broken attachment bonds. Relational trauma in infancy: psychoanalytic, attachment and neuropsychological contributions to parent–infant psychotherapy.

[49] Campbell F, Conti G, Heckman JJ, Moon SH, Pinto R, Pungello E (2014). Early childhood investments substantially boost adult health. Science.

[50] Bandura A (1977). Social learning theory. Social learning theory.

[51] Spence SH, Rapee RM (2016). The etiology of social anxiety disorder: an evidence-based model. Behav Res Ther.

[52] Barlow J, Bergman H, Kornør H, Wei Y, Bennett C. Group-based parent training programmes for improving emotional and behavioural adjustment in young children. Cochrane Database Syst Rev. 2016;8. 10.1002/14651858.CD003680.pub3 PMC6797064 27478983

[53] Moran P, Ghate D, Van Der Merwe A, bureau P research. What works in parenting support?: a review of the international evidence. Department for Education and Skills London; 2004.

[54] Barlow J, Coren E. The effectiveness of parenting programs: a review of Campbell reviews. Res Soc Work Pract [Internet]. 2017 22;28(1):99–102. Available from: https://doi.org/10.1177/1049731517725184.

[55] Loechner J, Starman K, Galuschka K, Tamm J, Schulte-Körne G, Rubel J, et al. Preventing depression in the offspring of parents with depression: a systematic review and meta-analysis of randomized controlled trials. Clin Psychol Rev [Internet]. 2018;60:1–14. Available from: http://www.sciencedirect.com/science/article/pii/S027273581730260X. 10.1016/j.cpr.2017.11.009 29305152

[56] Wyatt Kaminski J, Valle LA, Filene JH, Boyle CL. A meta-analytic review of components associated with parent training program effectiveness. J Abnorm Child Psychol [Internet]. 2008;36(4):567–89. Available from: https://doi.org/10.1007/s10802-007-9201-9. 10.1007/s10802-007-9201-9 18205039

[57] Filene JH, Kaminski JW, Valle LA, Cachat P (2013). Components associated with home visiting program outcomes: a meta-analysis. Pediatrics.

[58] Barlow J, Smailagic N, Huband N, Roloff V, Bennett C. Group-based parent training programmes for improving parental psychosocial health. Cochrane Database Syst Rev. 2014;5. 10.1002/14651858.CD002020.pub4 PMC10898322 24838729

[59] Yap MBH, Morgan AJ, Cairns K, Jorm AF, Hetrick SE, Merry S. Parents in prevention: a meta-analysis of randomized controlled trials of parenting interventions to prevent internalizing problems in children from birth to age 18. Clin Psychol Rev [Internet]. 2016;50:138–58. Available from: http://www.sciencedirect.com/science/article/pii/S0272735815301173. 10.1016/j.cpr.2016.10.003 27969003

[60] Mingebach T, Kamp-Becker I, Christiansen H, Weber L (2018). Meta-meta-analysis on the effectiveness of parent-based interventions for the treatment of child externalizing behavior problems. PLoS One.

[61] Barlow J, Smailagic N, Bennett C, Huband N, Jones H, Coren E (2011). Individual and group based parenting programmes for improving psychosocial outcomes for teenage parents and their children. Cochrane Database Syst Rev.

[62] Barlow J, Bennett C, Midgley N, Larkin SK, Wei Y (2015). Parent-infant psychotherapy for improving parental and infant mental health. Cochrane Database Syst Rev.

[63] Dretzke J, Davenport C, Frew E, Barlow J, Stewart-Brown S, Bayliss S (2009). The clinical effectiveness of different parenting programmes for children with conduct problems: a systematic review of randomised controlled trials. Child Adolesc Psychiatry Ment Health.

[64] Rayce SB, Rasmussen IS, Klest SK, Patras J, Pontoppidan M. Effects of parenting interventions for at-risk parents with infants: a systematic review and meta-analyses. BMJ Open [Internet]. 2017 1;7(12):e015707. Available from: http://bmjopen.bmj.com/content/7/12/e015707.abstract. 10.1136/bmjopen-2016-015707 PMC5770968 29284713

[65] McLeod BD, Weisz JR, Wood JJ (2007). Examining the association between parenting and childhood depression: a meta-analysis. Clin Psychol Rev.

[66] Rapee RM, Schniering CA, Hudson JL (2009). Anxiety disorders during childhood and adolescence: origins and treatment. Annu Rev Clin Psychol.

[67] Caldwell DM, Welton NJ (2016). Approaches for synthesising complex mental health interventions in meta-analysis. Evid Based Ment Health.

[68] Chaimani A, Caldwell DM, Li T, Higgins JPT SG. Undertaking network meta-analyses. In: Cochrane Handbook for Systematic Reviews of Interventions version 60 (updated July 2019) Cochrane, 2019. 2019.

[69] National Academies of Sciences and Medicine E. Parenting matters: supporting parents of children ages 0-8. National Academies Press; 2016. 27997088

[70] Moher D, Shamseer L, Clarke M, Ghersi D, Liberati A, Petticrew M (2015). Preferred reporting items for systematic review and meta-analysis protocols (PRISMA-P) 2015 statement. Syst Rev.

[71] Association AP. Diagnostic and statistical manual of mental disorders (DSM-5®). American Psychiatric Pub; 2013. 10.1590/s2317-17822013000200017 24413388

[72] Organization WH (1992). The ICD-10 classification of mental and behavioural disorders: clinical descriptions and diagnostic guidelines.

[73] Egger HL, Emde RN (2011). Developmentally sensitive diagnostic criteria for mental health disorders in early childhood: the diagnostic and statistical manual of mental disorders—IV, the research diagnostic criteria—preschool age, and the diagnostic classification of mental health. Am Psychol.

[74] Collishaw S, Sellers R. Trends in child and adolescent mental health prevalence, outcomes, and inequalities. Ment Heal Illn Child Adolesc. 2020:1–11.

[75] Peryer G, Golder S, Junqueira DR, Vohra S, Loke YK. Adverse effects. Cochrane Handb Syst Rev Interv. 2019:493–505.

[76] National Center for Parent F and CE. Compendium of parenting interventions. National Center on Parent, Family, and Community Engagement, Office of Head …; 2015.

[77] Stewart-Brown SL, McMillan AS (2010). Home and community based parenting support programmes and interventions: report of Workpackage 2 of the DataPrev project.

[78] Barlow J (2008). Health-led parenting interventions in pregnancy and early years.

[79] López-López JA, Welton NJ, Davies SR, Caldwell DM. Using network meta-analysis to identify effective components of complex mental health interventions. In: Research Synthesis 2019 incl Pre-Conference Symposium Big Data in Psychology, Dubrovnik, Croatia. ZPID (Leibniz Institute for Psychology Information); 2019.

[80] Ouzzani M, Hammady H, Fedorowicz Z, Elmagarmid A (2016). Rayyan—a web and mobile app for systematic reviews. Syst Rev.

[81] Collaboration C. Review Manager (RevMan) [Computer Program] Version 5.2. 3, The Nordic Cochrane Centre, Copenhagen, 2012. Health Psychol Rev. 2014;17.

[82] Systematic Review Data Repository. Accessed at https://srdr.ahrq.gov/.

[83] Deeks J, Higgins JPT, Altman DG. Analysing data and undertaking meta-analyses. Cochrane Handbook for Systematic Reviews of Interventions 5.0. 0. Assess July. 2011;15.

[84] Deeks JJ, Higgins JPT, Altman DG. Analysing data and undertaking meta-analyses. Cochrane Handb Syst Rev Interv Cochrane B Ser. 2008:243–96.

[85] Higgins JPT, Savović J, Page MJ, Elbers RG, Sterne JAC. Assessing risk of bias in a randomized trial. Cochrane Handb Syst Rev Interv. 2019:205–28.

[86] Mavridis D, Chaimani A, Efthimiou O, Leucht S, Salanti G (2014). Addressing missing outcome data in meta-analysis. Evid Based Ment Health.

[87] Faraone SV (2008). Interpreting estimates of treatment effects: implications for managed care. P T.

[88] Cohen J. Statistical power analysis for the behavioral sciences. 2nd. 1988;.

[89] Morris SB, DeShon RP (2002). Combining effect size estimates in meta-analysis with repeated measures and independent-groups designs. Psychol Methods.

[90] Morris SB (2008). Estimating effect sizes from pretest-posttest-control group designs. Organ Res Methods.

[91] Higgins JPT, Green S. Cochrane handbook for systematic reviews of interventions. Vol. 4. John Wiley & Sons; 2011.

[92] López-López JA, Page MJ, Lipsey MW, Higgins JPT (2018). Dealing with effect size multiplicity in systematic reviews and meta-analyses. Res Synth Methods.

[93] Jakobsen JC, Gluud C, Wetterslev J, Winkel P (2017). When and how should multiple imputation be used for handling missing data in randomised clinical trials – a practical guide with flowcharts. BMC Med Res Methodol.

[94] Jakobsen JC, Gluud C, Winkel P, Lange T, Wetterslev J (2014). The thresholds for statistical and clinical significance–a five-step procedure for evaluation of intervention effects in randomised clinical trials. BMC Med Res Methodol.

[95] Jakobsen JC, Wetterslev J, Winkel P, Lange T, Gluud C (2014). Thresholds for statistical and clinical significance in systematic reviews with meta-analytic methods. BMC Med Res Methodol.

[96] Davies SR, Caldwell DM, Lopez-Lopez JA, Dawson S, Wiles N, Kessler D, et al. The process and delivery of cognitive behavioural therapy (CBT) for depression in adults: a network meta-analysis. Cochrane Database Syst Rev. 2018;10.

[97] CHEN Y, ZHANG J (2012). Guideline on missing data in confirmatory clinical trials. Chinese J New Drugs.

[98] Freeman SC, Scott NW, Powell R, Johnston M, Sutton AJ, Cooper NJ. Component network meta-analysis identifies the most effective components of psychological preparation for adults undergoing surgery under general anaesthesiaonline only appendix present. J Clin Epidemiol. 2018;. 10.1016/j.jclinepi.2018.02.012 29476923

[99] Chaimani A, Caldwell DM, Li T. Chapter 11: Undertaking network meta-analyses: Higgins JPT, Thomas J, Chandler J, et al., Cochrane Handbook for Systematic Reviews of Interventions. version 6.0 (updated July 2019) Cochrane, 2019.

[100] Bucher HC, Guyatt GH, Griffith LE, Walter SD (1997). The results of direct and indirect treatment comparisons in meta-analysis of randomized controlled trials. J Clin Epidemiol.

[101] Dias S, Ades AE, Welton NJ, Jansen JP, Sutton AJ. Network meta-analysis for decision-making. John Wiley & Sons; 2018.

[102] Sterne JAC, Sutton AJ, Ioannidis JPA, Terrin N, Jones DR, Lau J (2011). Recommendations for examining and interpreting funnel plot asymmetry in meta-analyses of randomised controlled trials. BMJ..

[103] Higgins JPT, Altman DG, Sterne JAC. Chapter 8: assessing risk of bias in included studies. Higgins JPT, Green S (eds). Cochrane Handbook for Systematic Reviews of Interventions. Version 5.1. 0. Cochrane Collaboration. 2016.

[104] Boutron I, Page MJ, Higgins JPT, Altman DG, Lundh A, Hróbjartsson A, et al. Considering bias and conflicts of interest among the included studies. Cochrane Handb Syst Rev Interv. 2019:177–204.

[105] Richardson M, Garner P, Donegan S. Interpretation of subgroup analyses in systematic reviews: a tutorial. Clin Epidemiol Glob Heal. 2018.

[106] Salanti G, Del Giovane C, Chaimani A, Caldwell DM, Higgins JPT. Evaluating the quality of evidence from a network meta-analysis. PLoS One [Internet]. 2014 3;9(7):e99682. Available from: https://doi.org/10.1371/journal.pone.0099682. 10.1371/journal.pone.0099682 PMC4084629 24992266

[107] Guyatt GH, Oxman AD, Schünemann HJ, Tugwell P, Knottnerus A (2011). GRADE guidelines: a new series of articles in the journal of clinical epidemiology. J Clin Epidemiol.

[108] Nikolakopoulou A, Higgins JPT, Papakonstantinou T, Chaimani A, Del Giovane C, Egger M, et al. Assessing confidence in the results of network meta-analysis (CINeMA). bioRxiv. 2019;597047. 10.1371/journal.pmed.1003082 PMC7122720 32243458

[109] Phillippo DM, Dias S, Welton NJ, Caldwell DM, Taske N, Ades AE. Threshold analysis as an alternative to GRADE for assessing confidence in guideline recommendations based on network meta-analyses. Ann Intern Med [Internet]. 2019/03/26. 2019 16;170(8):538–46. Available from: https://pubmed.ncbi.nlm.nih.gov/30909295. 10.7326/M18-3542 PMC6739230 30909295

[110] Roza SJ, Hofstra MB, van der Ende J, Verhulst FC. Stable prediction of mood and anxiety disorders based on behavioral and emotional problems in childhood: a 14-year follow-up during childhood, adolescence, and young adulthood. Am J Psychiatry [Internet]. 2003 1;160(12):2116–21. Available from: https://doi.org/10.1176/appi.ajp.160.12.2116. 10.1176/appi.ajp.160.12.2116 14638580

[111] Kwong ASF, Manley D, Timpson NJ, Pearson RM, Heron J, Sallis H (2019). Identifying critical points of trajectories of depressive symptoms from childhood to young adulthood. J Youth Adolesc.

[112] von Klitzing K, White LO, Otto Y, Fuchs S, Egger HL, Klein AM (2014). Depressive comorbidity in preschool anxiety disorder. J Child Psychol Psychiatry.

[113] McElroy E, Fearon P, Belsky J, Fonagy P, Patalay P (2018). Networks of depression and anxiety symptoms across development. J Am Acad Child Adolesc Psychiatry [Internet].

[114] Dyer ML, Heron J, Hickman M, Munafò MR. Alcohol use in late adolescence and early adulthood: the role of generalized anxiety disorder and drinking to cope motives. Drug Alcohol Depend [Internet]. 2019;107480. Available from: http://www.sciencedirect.com/science/article/pii/S037687161930239X. 10.1016/j.drugalcdep.2019.04.044 PMC6891250 31706711

[115] Costello EJ, Mustillo S, Erkanli A, Keeler G, Angold A. Prevalence and development of psychiatric disorders in childhood and adolescence. Arch Gen Psychiatry [Internet]. 2003 1;60(8):837–44. Available from: https://doi.org/10.1001/archpsyc.60.8.837. 10.1001/archpsyc.60.8.837 12912767

